# Supplementary material for: Switching nanoprecipitates to resist hydrogen embrittlement in high-strength aluminum alloys
Source: Nat Commun. 2022 Nov 18;13:6860. doi: 10.1038/s41467-022-34628-4 (PMC9674592; doi:10.1038/s41467-022-34628-4)
Supplement: Supplementary file 1 — Supplementary Information [file 41467_2022_34628_MOESM1_ESM.pdf]

Supplementary Materials for

Switching nanoprecipitates to resist hydrogen embrittlement in high-strength aluminum alloys

Yafei Wang, Bhupendra Sharma, Yuantao Xu, Kazuyuki Shimizu, Hiro Fujihara, Kyosuke Hirayama, Akihisa Takeuchi, Masayuki Uesugi, Guangxu Cheng, Hiroyuki Toda

Correspondence to: yafeiwang90@outlook.com; sharma.bhupendra.464@m.kyushu-u.ac.jp; xu.yuantao.158@m.kyushu-u.ac.jp.

Supplementary Table 1. Effect of ZPE on the calculated hydrogen trap energies at T phase.

Site	Trap energy (eV/atom)	
	With ZPE	Without ZPE
A	0.603	0.579
B	0.556	0.539
C	0.338	0.31
D	0.305	0.293
E	0.239	0.215
F	0.192	0.175
G	0.133	0.129
H	0.005	-0.041
I	-0.055	-0.075
J	-0.104	-0.13
K	-0.165	-0.195
L	-0.194	-0.226

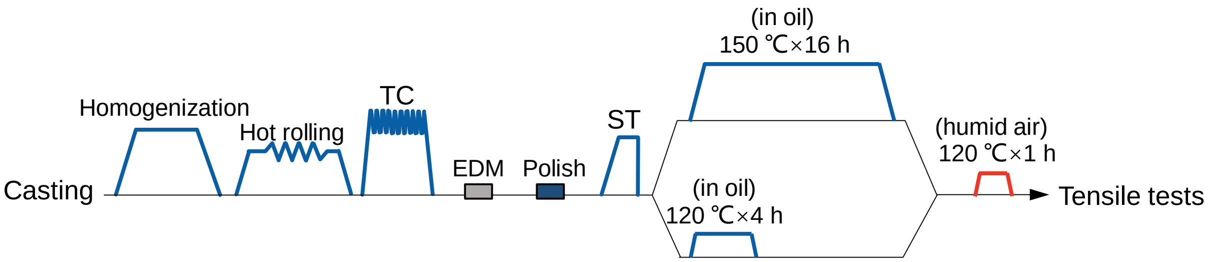

Supplementary Fig. 1. Illustration of the specimen preparation procedures. Aging temperature was elevated from 120 to 150 °C to induce T-phase precipitation. Hydrogen charging was done mainly by aging in humid air, although hydrogen ingress is also expected to occur during EDM cutting in water.

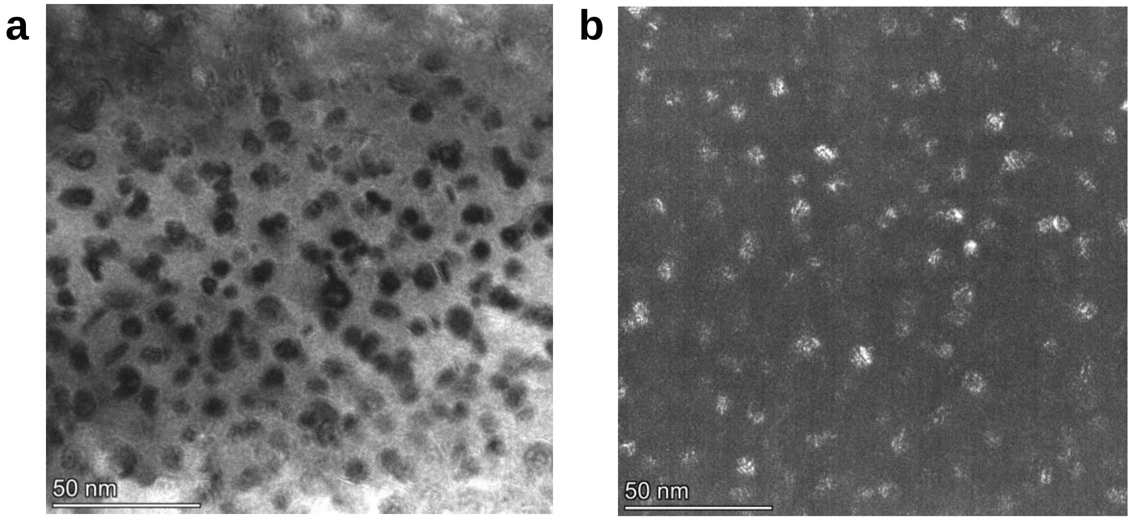

Supplementary Fig. 2. TEM confirmation of the fraction of T phase. **a** Bright-field TEM image of the precipitates and **b** dark-field TEM images of the T phase in the same position along the $[110]_{Al}$ zone axis, based on which the fraction of T phase was estimated to be at least 50%.

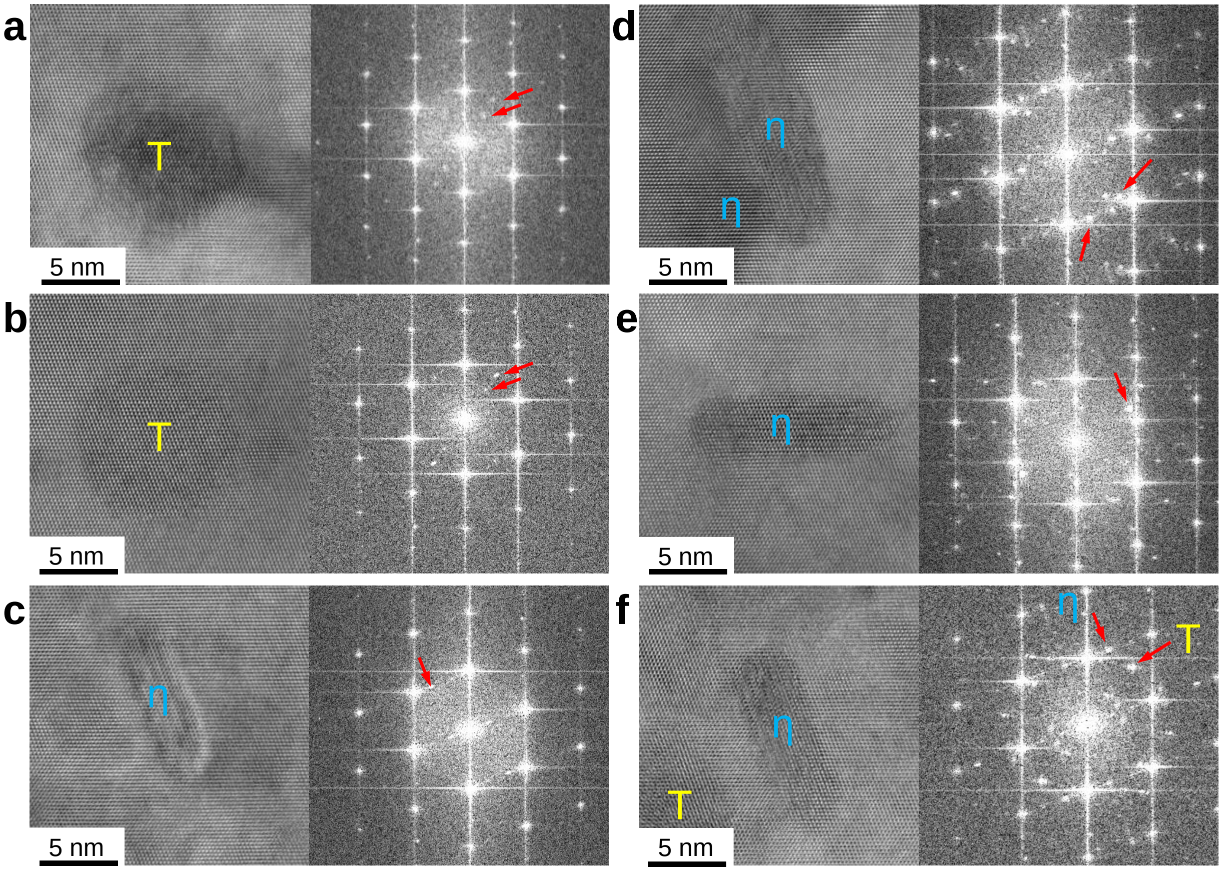

Supplementary Fig. 3. HAADF-STEM images and fast Fourier transform analyses of precipitates in HT material. **a** and **b** T phase, **c-e** η phase, **f** co-existence of T and η. Images were taken along the $[110]_{Al}$ zone axis.

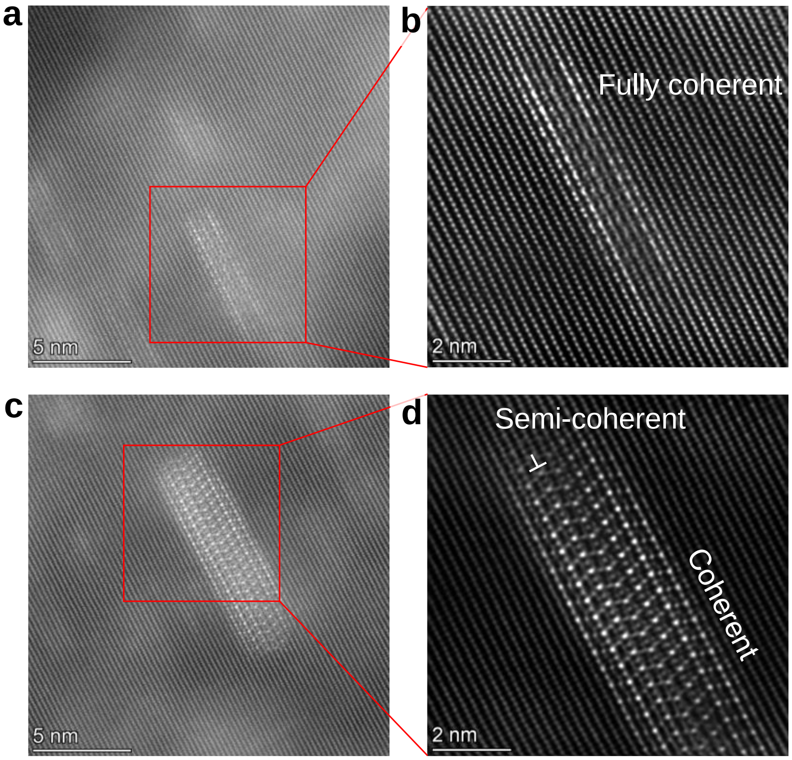

Supplementary Fig. 4. HAADF-STEM images of fine and coarse η precipitates for the confirmation of interfacial coherency. **a** HAADF image of a fine η phase with a diameter of 5.5 nm and a height of 1.5 nm, and **b** enlarged image showing fully coherent interfaces; **c** HAADF image of a coarse η phase with a diameter of 10.6 nm and a height of 2.6 nm, and **d** enlarged image showing semi-coherent interfaces at the edges.

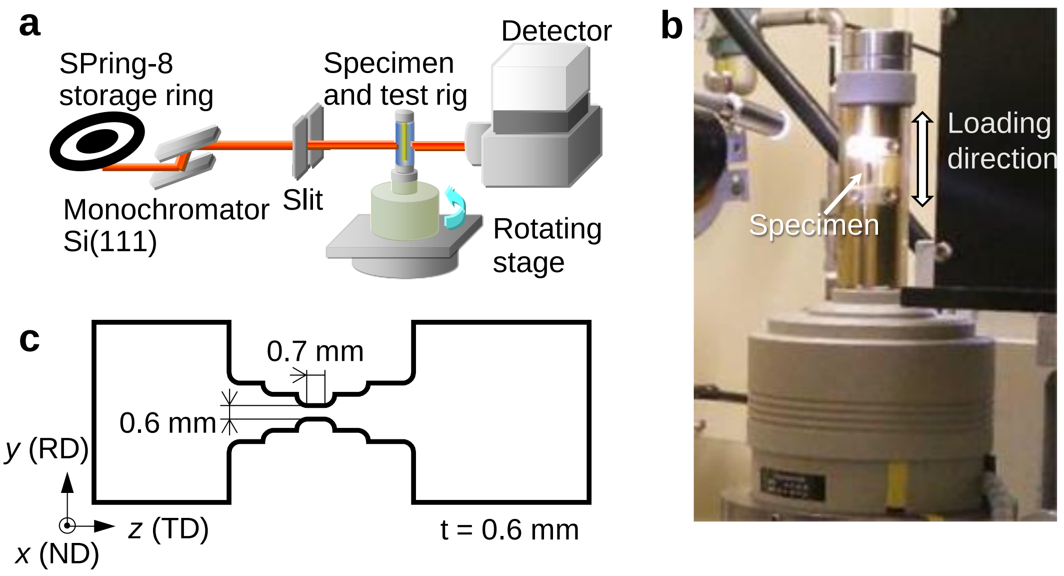

Supplementary Fig. 5. Illustration of the experimental setup. **a** synchrotron radiation facility. **b** setup of in-situ tensile test. **c** geometry of the tensile specimen.

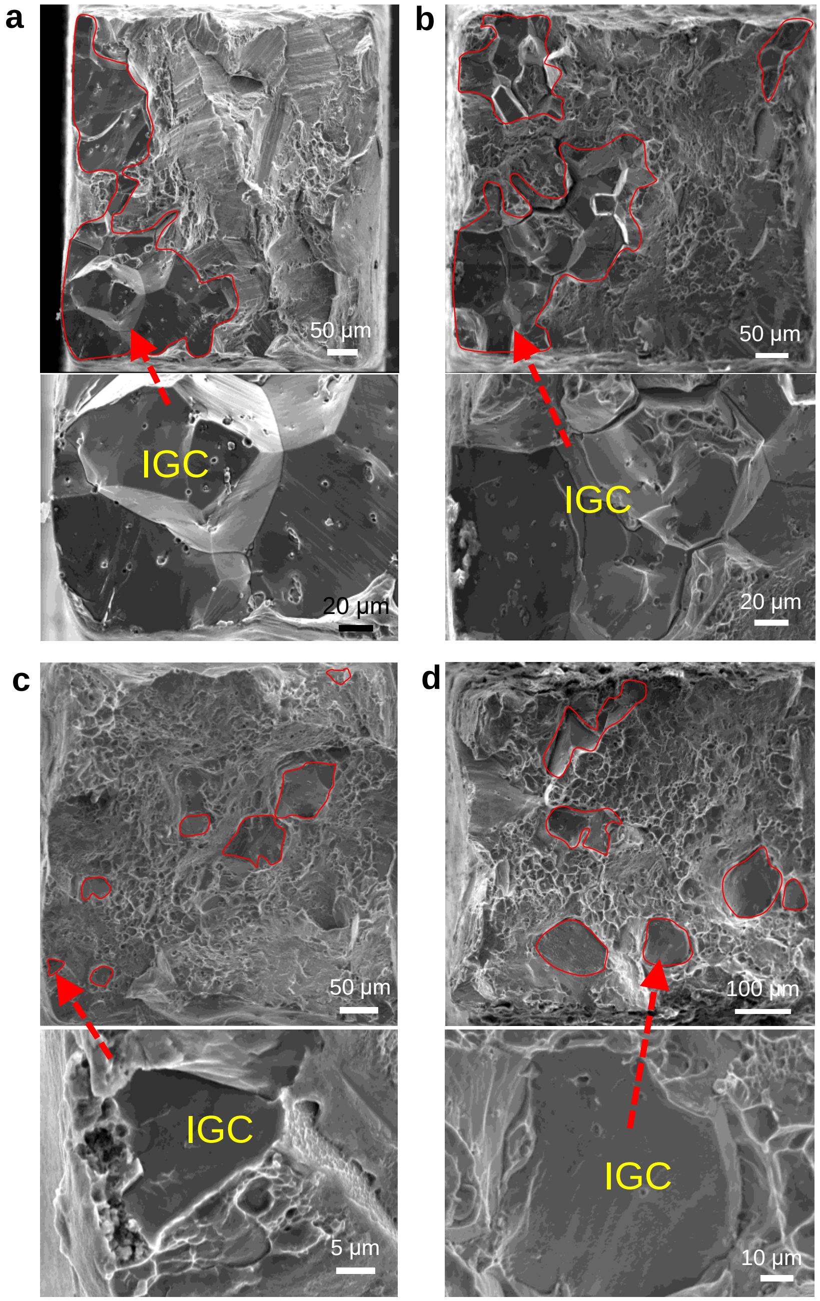

Supplementary Fig. 6. Typical morphologies of IGCs obtained from repeat tensile tests. **a** and **b** SEM images of the fracture surfaces of LT specimens with the boundaries of IGC marked in red lines and its enlarged morphologies shown in the lower figures. **c** and **d** SEM images of the fracture surfaces of HT specimens with the boundaries of IGCs marked in red lines and its enlarged morphologies shown in the lower figures.

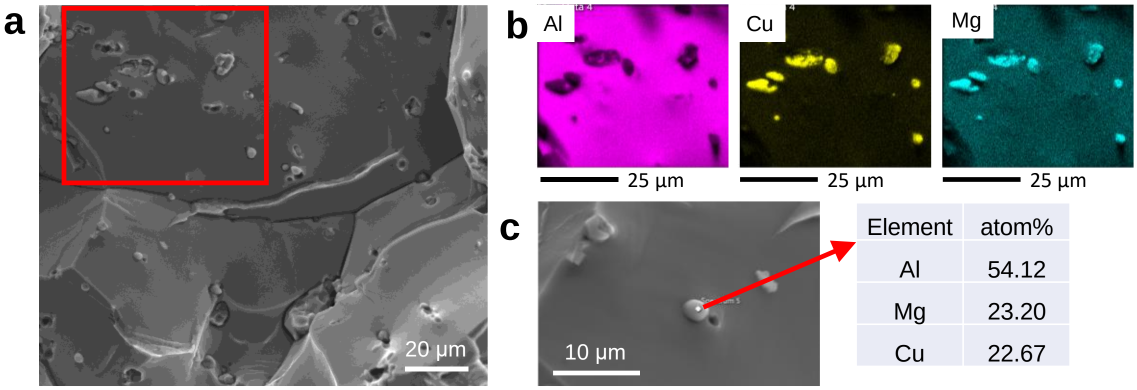

Element	atom%
Al	54.12
Mg	23.20
Cu	22.67

Supplementary Fig. 7. Confirmation of S phase particles. **a** SEM image of the intermetallic particles at grain boundaries on the fracture surface. **b** EDS mapping of the region marked in **a**. **c** enlarged morphology of particles and the corresponding elemental composition obtained from point EDS analysis, confirming the particles as S phase.
